# Novel antibiotic resistance profiles in bacteria isolated from oil fly larvae *Helaeomyia petrolei* living in the La Brea Tar Pits

**DOI:** 10.1007/s10482-024-02050-z

**Published:** 2024-12-24

**Authors:** Lisa M. Durso, Md. Shamimuzzaman, Brian Dillard, Kenneth W. Nickerson

**Affiliations:** 1 USDA-ARS, 137 Keim Hall, 251 Filley Hall, Lincoln, NE 68583 USA; 2 School of Biological Sciences, University of Nebraska, Lincoln, NE 68588-0666 USA; 3 Present Address: Department of Ecology and Evolutionary Biology, Cornell University, Ithaca, NY USA

**Keywords:** Antibiotic resistance, Oil fly, La Brea, Efflux pumps, Extreme environment, Hydrocarbon, *Helaeomyia petrolei*

## Abstract

Larvae from the petroleum oil fly, *Helaeomyia petrolei*, live in the asphaltene and polyaromatic hydrocarbon rich asphalt seeps of Rancho La Brea, Los Angeles, California. These larvae pass high amounts of viscous asphalt through their digestive system, and their gut microbiota is exposed to these extreme conditions. Environmental stress response mechanisms can co-select for antibiotic resistance, and in the current study we used 16S rRNA and genomic sequencing along with the Comprehensive Antibiotic Resistance Database (CARD) tools to characterize antibiotic resistance profiles from six bacteria previously isolated from the oil fly larval intestinal tract, linking phenotypic and genotypic resistance profiles. The isolates contain a core set of antibiotic resistance determinants along with determinants that are rarely found in these species. Comparing these oil fly isolates to the phenotypic prevalence data generated by the CARD Resistance Gene Identifier revealed sixteen instances where the oil fly bacteria appeared to carry a resistance not seen in related taxa in the database, suggesting a novel suite of resistance families in the oil fly isolates compared to other members of the same taxa. Results highlight the functional duality of genes that simultaneously code for antibiotic resistance and survival under extreme conditions, and expand our understanding of the ecological and evolutionary role of antibiotic resistance genes in environmental habitats.

**Supplementary Information:**

The online version contains supplementary material available at 10.1007/s10482-024-02050-z.

## Introduction

Widespread use of antibiotics to treat infectious disease began in 1945 and revolutionized global health (Aminov, 2010), increasing the average U.S. lifespan by 23.6 years between 1920 and 2015 (Ventola, 2015). The initial success of this approach has now been tempered by the development and spread of numerous mechanisms whereby microbes have become antibiotic resistant. This competition has become so intense that there are serious concerns about the appearance of microbes which are impervious to all known antibiotics. Although antibiotic resistant bacteria and antibiotic resistance genes occur naturally and can be found in ancient and remote samples (Hobson et al. 2021, Larsen et al. 2022), their prevalence has increased rapidly since the introduction of antibiotic drug therapy (Gillings and Paulsen, 2014; Perry et al. 2016). The rapidity with which this phalanx of antibiotic resistance genes has appeared has spurred international collaborative efforts to address antibiotic resistance. The World Health Organization (WHO) Global Action Plan on Antimicrobial Resistance (2015) aims to maintain the ability to treat and prevent infectious diseases through a multi-pronged approach that includes understanding the ecology and evolution of antibiotic resistant bacteria and antibiotic resistance genes, while simultaneously strengthening efforts to develop new medicines.

Microbial diversity in extreme habitats is the foundation for new strategies to address antibiotic resistance (Sayed et al. 2020), as well as the source of bacteria harboring novel antibiotic resistance genes (Yi et al. 2022; Marcoleta et al. 2022). The environment in which bacteria live impacts the evolution of not only antibiotic resistance, but also a myriad of functional metabolites and bioactive compounds (Larsson and Flach, 2022). Innovative approaches are emerging that combine next generation DNA -based sequencing tools with novel biochemical and bio-molecular techniques to probe the ecology of antibiotic resistance and develop new therapeutic products (Hobson et al. 2021, Sayed et al. 2020). Harsh abiotic conditions are a driver for the development of bacteria with novel bioactive metabolites and enzyme pathways (Merino et al. 2019; Kohli et al. 2020; Sayed et al. 2020) and extreme habitats, such as the La Brea Tar Pits, provide unique selective pressures for the organisms they harbor.

The La Brea Tar Pits are a collection of naturally occurring hydrocarbon asphaltene seeps in Southern California, surrounded by urban Los Angeles. They are formed from viscous heavy oil and asphaltenes that ooze up from the underground Salt Lake Oil Field through cracks in the earth (McNassor, 2011; O’Rilley 2021). This asphalt is known to be composed of a wide variety of aromatic hydrocarbons including substances like toluene and anthracene. Many of these compounds readily pass through cellular membranes and thus can be highly toxic (Sikkema et al. 1995). The modern-day site remains dynamic, with shifting tar sands and the development of new pools of asphalt. The asphalt “tar” pits (“brea” is Spanish for tar) are well known for the many large Pleistocene mammal fossils unearthed from the site, and for the Native American Chumash and Tongva People of the Greater Los Angeles Basin who pioneered uses of the La Brea tar-based asphalt for waterproofing, tools, and repair (Braje et al. 2005). Less well known are the modern-day invertebrate, bacterial and archaeal species that survive and grow in these recalcitrant hydrocarbon matrices (Kadavy et al. 1999; Kim and Crowley, 2007).

One remarkable inhabitant of the La Brea Tar Pits is the oil fly (***Helaeomyia petrolei***). The larvae were first observed in oil fields shortly after the dawn of the petroleum industry (Peckham, 1894), and the organism was later characterized by Thorpe (1930, 1931). Thorpe referred to the oil fly as “undoubtedly one of the chief biological curiosities of the world” in that the carnivorous larvae are exclusively found submerged in oil, where they ingest large quantities of oil and asphalt without suffering any ill effects (Thorpe 1930, 1931). The larvae feed on dead and dying insects found in the tar, while simultaneously ingesting high amounts of asphalt through their digestive system. They do not derive nutrients from the asphalt but can survive and grow by feeding in this harsh environment (Rotheray, 2019).

Oil flies have a complex gut flora (Nickerson and Plantz, 2017; Kim and Crowley, 2007) which is perforce in contact with the asphalt. Previously, culture-based approaches were used to isolate bacteria from the oil fly gut (Kadavy et al. 1999), and characterize their antibiotic resistance phenotypes for a subset of targets (Kadavy et al. 2000). Although culture-based antibiotic susceptibility testing (AST) remains the gold standard in clinical laboratories due to the ability of linking antibiotic resistance carriage to host genomic background (McLain et al. 2016), AST methods are time and labor intensive, require expensive reagents, and provide information on only a limited number of targets. Molecular based whole genome sequencing (WGS) is increasingly being used to characterize the full suite of antibiotic resistance genes in any given isolate, due to its ability to simultaneously detect all known resistance determinants in a single physical test (Ellington et al. 2017; Su et al. 2019; Coll et al. 2024). In this context, we hypothesize that the DNA sequence data from these oil fly bacteria will reveal a unique complement of antibiotic resistance genes associated with survival in the tar.

## Materials and methods

### **Sample collection, processing, and strain verification**

Oil fly larvae and asphalt were previously collected from the La Brea Tar Pits in Southern California and their bacterial intestinal contents processed and characterized as described by Kadavy et al. (1999). Of the 14 original isolates stored continuously in glycerol at  − 80 °C, 13 were recovered and six (Oil Fly (OF) 2, 4, 5, 6, 8 and 10) were used for the current WGS antibiotic resistance gene evaluation. These isolates were chosen to represent the breadth of species available in the original set, with OF10 providing variation within a single species. The multiple *Providencia* (genus) isolates were not clonally related in that they had previously given different identities by their fatty acid profiles and BBL Enterotube II analyses (Kadavy et al. 1999). OF2 was not included among the antibiotic resistance patterns identified previously (Kadavy et al. 2000) and this omission was corrected by testing OF2 against 18 antibiotics (Dillard, 2020). All isolates were plated on Luria Bertani (LB) Agar (Difco, Franklin Lakes, NJ) and grown overnight at 37 °C. Plates were evaluated for purity and morphological features were confirmed before a single colony was selected for overnight growth in LB Broth (at 37 °C) for DNA harvest.

Genomic DNA was obtained from 0.25 mL of the overnight culture for each isolate with the QIA-Amp Power Fecal DNA kit (product number 12830; Qiagen Inc., Germantown, MD), used according to the manufacturer’s instructions. To confirm isolate identity via their 16 s rRNA sequences, extracted genomic DNA from the 13 OF isolates was overnight shipped to Molecular Research LP (Shallowater, TX). A 35-cycle PCR was performed using 27F and 1492R primers and the HotStar Taq Plus Master Mix (Qiagen, Germantown, MD). DNA quality was checked using a 2% agarose gel and purified using Ampure PB beads (Pacific Biosciences, Menlo Park, CA). Libraries were created using the SMRTbell library kit from Pacific Biosciences (Menlo Park, CA) and sequenced on a PacBio Sequel following the manufacturer’s guidelines.

Data was processed using the PacBio Circular Consensus Sequencing algorithm to merge overlapping forward and reverse reads. Data was processed using a proprietary analysis pipeline (www.mrdnalab.com, MR DNA, Shallowater, TX) that began by removing barcodes, primers, sequences with ambiguous base calls or homopolymer runs exceeding 6 bp, and sequences less than 200 bp in length. Sequences were denoised followed by removal of chimera and singleton sequences. Next, Operational taxonomic units were defined (97% similarity), and classified using BLASTN (BLAST + v2.1.5.0) against a curated GreenGenes (Greengenes2 2022.10) database (DeSantis et al. 2006). All bioinformatics analysis was performed using USDA-ARS high-performance computing (HPC) clusters named SCINet.

### **Genome sequencing of the selected OF strains and initial data processing**

On the basis of 16S rRNA sequencing and identification, six OF strains (OF2, OF4, OF5, OF6, OF8, OF10) were selected for WGS. Pelleted cells of oil fly gut isolates were shipped overnight for extraction and sequencing, performed by The Sequencing Center (Fort Collins, CO). WGS libraries were prepared using the Illumina Nextera XT Library Kit and Nextera XT Index Kit (Illumina, San Diego, CA), and sequenced on a MiSeq sequencing instrument to generate 2 X 250 bp paired end reads. Demultiplexing was performed using Illumina provided software named bcl2fastq (v2.20.0.422. Data files were initially quality checked using the FastQC (v0.12.1) tools. Adapter trimming and low-quality reads were removed to generate clean FastQ files using Trimmomatic v0.38 software (Bolger et al. 2014). Scripts (Trimmomatic.sh and FastQC.sh) that were used to perform quality checking and adapter trimming are available at https://github.com/shamim-ars/ARG_Identification.

### **WGS data mapping to 16S rRNA sequence**

To corroborate the 16S rRNA sequencing results, we mapped WGS reads to the individual 16S rRNA sequence separately using Hisat2 v2.2.1 aligner (Kim et al. 2019) with default settings for paired end reads. Then we used sequence alignment/map (SAM v1.17) tools (Danecek et al. 2021) to convert the SAM files into binary alignment map (BAM) files. Finally, we visualized the reads Pileup (format) against the reference 16S rRNA sequence for each sample using Integrative Genomics Viewer (IGV) v2.17.4 (Robinson et al. 2011). Additionally, we retrieved the consensus sequence from the aligned reads and performed a BLAST (BLAST + v2.15.0) search against the individual 16S rRNA sequences to check the percent identity. All 16 s rRNA sequences obtained as part of this study have been deposited in NCBI (Table 1). Scripts that were used to perform alignment (Hisat2.sh) and file conversion (Samtools_file_conversion.sh) are available at https://github.com/shamim-ars/ARG_Identification.

**Table 1:** Overview of the classification and antibiotic resistance annotations of bacterial isolates originally cultured from the oil fly (Kadavy et al. 1999). The oil fly bacterial isolates selected for this study are indicated with an asterisk (*)

Strain #^*^	Original (1999) classification	16S classification	# of AR^§^ pheno-types (n = 23)	NRRL culture collection #	NCBI accession #
OF1	*Providencia rettgeri*	*Providencia vermicola*	12	B-65562	MW466638
OF2*	Inconclusive^b^	Uncultured *Alcaligenes sp.*	8^‡^	B-65563	MN527032
OF4*	*Pseudomonas maltophila*^*†*^	*Pseudomonas aeruginosa*	NA	NA	MN547155
OF5*	*Providencia rettgeri*	*Providencia vermicola*^*d*^	11	B-65564	MN547314
OF6*	*Providencia rettgeri*	*Providencia rettgeri*	10	B-65565	MN547624
OF7	*Providencia rettigeri*	*Providencia vermicola*	12	B-65566	MW466640
OF8*	*Morganella morganii*	*Morganella morganii*	10	B-65567	MN547625
OF9	*Providencia rettgeri*	*Providencia rettgeri*	9	B-65568	MW466642
OF10*	*Providencia rettgeri*	*Providencia vermicola*	11	B-65569	MN547993
OF11	*Providencia rettgeri*	*Providencia vermicola*	11	B-65570	MW466641
OF12	*Providencia rettgeri*	*Providencia vermicola*	12	B-65571	MW466695
OF13	Inconclusive	Uncultured *Providencia sp.*	8	B-65572	MW466643
OF14	*Providencia rettgeri*	*Providencia vermicola*	11	B-65573	MW466696

*Strain numbers and original classifications are from (Kadavy et al. 1999), while antibiotic resistant (AR) phenotypes are from (Kadavy et al. 2000); strain OF3 was no longer viable after 20 years storage at  − 80 °C. † Both OF2 and OF4 were classified as nonenteric by API 20E and as either *Acinetobacter lwoffi* or *Pseudomonas maltophila* by Enterotube. ‡ OF2 is resistant to 8 of 17 antibiotics (Dillard, 2020 (MS Thesis)) while the other strains are resistant to 8–12 of 23 antibiotics (Kadavy et al. 2000). All bacteria are available from the Agricultural Research Service Culture Collection (NRRL) https://nrrl.ncaur.usda.gov/. ^§^ Antibiotic resistance

### **Identification of antibiotic resistance genes from WGS datasets**

Identification of antibiotic resistance genes was carried out using the Comprehensive Antibiotic Resistance Database (CARD v3.2.8)-Resistance Gene Identifier (RGI v6.0.2) bioinformatic pipeline (Alcock et al. 2023), which provides genotype analysis and phenotype prediction. Adapter trimmed, cleaned, FastQ paired end reads were aligned to reference sequences from CARD's protein homolog models, protein variant models, rRNA mutation models, and protein over-expression models. Default parameters were used for running the CARD Resistance Gene Identifier (RGI) Burrows-Wheeler transform (bwt) function with FastQ files for all model types. We used RGI bwt read mapping results at the gene level for further analysis. The CARD database was used for this analysis because of its expertly curated and updated database, high number of AMR reference genes, ability to identify mutations conferring resistance, and flexibility of the RGI annotation interface. The script that was used to perform antibiotic resistance gene identification is available at https://github.com/shamim-ars/ARG_Identification/blob/main/CARD_RGI.sh.

### **Comparison of oil fly isolate profiles**

In order to contextualize the antibiotic resistance genotype profiles obtained from the oil fly isolates, they were compared to results from the same or similar species isolated from other sources. The CARD Phenotypic Prevalence database was used for this analysis. The database compiles completely sequenced genomes and plasmids, whole-genome shotgun assemblies, and sequenced genomic islands, and inventories them for presence of antimicrobial resistance determinants that are associated with the CARD phenotypic class, as defined by the CARD antibiotic resistance ontology (ARO) phenotypic classification and scored by the Resistance Gene Identifier (RGI). The result is a percentage-based measure of prevalence. For the current analysis (run 12-Feb-2024) the CARD analysis was based on 625 chromosomes, 342 plasmids, and 7019 WGS for *Pseudomonas aeruginosa*; 52 chromosomes, 40 plasmids, and 163 WGS for *Morganella morganii*; and 34 chromosomes, 37 plasmids, and 157 WGS for *Providencia rettgeri*. Data for *Provedencia vermicola* were not available in the database. However, recent genomic analysis commonly groups *P. vermicola* with *P. rettgeri* (Andolfo et al. 2021)*,* so for the current analysis all three *Providencia* isolates were compared to the CARD data for *P. rettgeri.* In absence of species information for OF2, it was compared to *Alcaligenes faecalis*, which had 20 chromosomes, 5 plasmids, and 24 WGS in the CARD phenotypic profile database.

### **Strain accessibility**

All 13 oil fly isolates were deposited into the Agricultural Research Service Northern Regional Research Laboratory (NRRL) United States Department of Agriculture bacterial culture collection in Peoria, IL. Strains are available for research purposes via https://nrrl.ncaur.usda.gov/.

## Results

### **Strain verification and WGS data mapping to 16S rRNA sequence**

Twenty years after they were originally isolated, we re-examined the oil fly gut bacteria using modern sequencing techniques (Table 1). With the exception of OF2 and OF013, the identifications by 16S sequencing confirmed or closely approximated those obtained previously by the BBL Enterotube II and API 20E systems and fatty acid analysis (Kadavy et al. 1999). With more complete sequence databases amassed over the last 20 years, the two isolates previously identified as *Providencia rettgeri* were now recognized as *Providencia vermicola* and the isolate originally identified as *Pseudomonas maltophila* is now identified as *Pseudomonas aeruginosa* (Table 1). Six isolates were chosen for WGS. Because of known potential biases associated with 16S rRNA sequencing, we confirmed the 16S rRNA results by aligning the WGS reads and comparing identity (Table 2).

**Table 2:** Summary of 16S rRNA Sequencing matching with WGS datasets

Sample Name	NCBI WGS Accession	16S rRNA Accession	Percent ID of WGS reads aligned to 16S rRNA sequence (%)	Best matched species (WGS)	Classification based on 16S alone
OF2	SRR29058172	MN527032	100	*Alcaligenes sp.*	Uncultured *Alcaligenes sp.*
OF4	SRR29058171	MN547155	100	*Pseudomonas aeruginosa*	*Pseudomonas aeruginosa*
OF5	SRR29058170	MN547314	99.81	*Providencia vermicola*	*Providencia vermicola*^*d*^
OF6	SRR29058169	MN547624.1	100	*Providencia rettgeri*	*Providencia rettgeri*
OF8	SRR29058168	MN547625	100	*Morganella morganii*	*Morganella morganii*
OF10	SRR29058167	MN547993	99.94	*Providencia vermicola*	*Providencia vermicola*

### **Identification of antibiotic resistance genes from WGS datasets**

The six sequenced oil fly isolates collectively carried genes coding for resistance to 29 of the 49 drug classes in the CARD database (range 20–22 per isolate), and 48 of 308 antibiotic resistance gene families found in the database (range 30–81 per isolate), with 170 total antibiotic resistance genes identified across the five species (*two P. vermicola*). (Table 3, Supplementary Tables S1–S7). As part of the analysis, antibiotic resistance genes or determinants in the oil fly larval isolates that matched a gene or determinant in CARD were extracted and concurrently assigned to the relevant gene families and drug classes. For example, OF2 has 2258 reads classified as aminoglycoside resistance. Of these, six work via inactivation, 532 via efflux and 1720 via inactivation. For that same drug class (aminoglycosides) OF4 has 5019 total reads, 1029 inactivation, 1387 efflux, and 2602 target alteration, organized into 11 gene families. Of the 29 drug classes identified in our sample set, 16/29 were represented in all five oil fly isolates, and 6/29 occurred in only a single isolate. OF4, the *Pseudomonas aeruginosa* isolate, had the highest number of mapped reads assigned, both for drug classes (21 of 29) and gene families (n = 81).

**Table 3:** The six oil fly larvae bacterial isolates characterized in this study had genes coding for resistance to 29 drug classes, listed here. Each drug class was represented by at least one antibiotic resistance gene family and mechanism. For example, OF2 has genes classified into three different aminoglycoside antibiotic gene families (efflux, inactivation, and target alteration). OF4 has genes classified into 11 gene families, with the same three mechanisms of action. See Supplementary data files S1–S6 for complete gene family list for each isolate

	AR* mechanism							**# of gene families**					
**Drug class**	Efflux	Inactivation	Target Alt	Reduce Perm	Absence	Target Replace	Target Protect	**OF2** *Alcaligenes sp.*	**OF4** *P. aeruginosa*	**OF5** *P. vermicola*	**OF6** *P. rettgeri*	**OF8** *M. morganii*	**OF10** *P. vermicola*
Aminocoumarin antibiotic	a							1	1	1	1	2	1
Aminoglycoside antibiotic	a	b	c					3	11	4	5	7	6
Bicyclomycin-like abx	a							0	1	0	0	0	0
Carbapenem	a	b	c	d	e			1	4	3	3	3	2
Cephalosporin	a	b	c	d	e			1	6	6	7	7	5
Cephamycin	a	b	c	d	e			1	0	4	2	4	2
Diaminopyrimidine abx	a		c					0	1	1	1	1	1
Elfamycin antibiotic			c					1	1	1	1	1	1
Fluoroquinolone abx	a		c	d	e			5	9	7	7	9	7
Fusidane antibiotic			c					1	0	0	0	0	0
Glycopeptide antibiotic	a		c					1	4	4	4	0	5
Glycylcycline	a		c					0	3	0	1	1	0
Isoniazid-like antibiotic			c					0	0	0	0	1	0
Lincosamide antibiotic			c					1	1	1	1	1	1
Macrolide antibiotic	a		c	d	e			3	6	4	4	5	4
Monobactam	a	b	c	d	e			1	3	1	1	2	1
Nitroimidazole antibiotic	a							0	0	0	0	1	1
Nucleoside antibiotic	a							0	0	0	0	0	1
Oxazolidinone antibiotic	a							1	2	1	1	1	1
Pen**a**m	a	b	c	d	e			2	5	5	5	5	4
Pen**e**m	a	b			e			2	3	3	4	3	3
Peptide antibiotic	a	b		d	e			1	4	5	3	6	3
Phenicol antibiotic	a	b	c	d	e			1	5	2	2	4	2
Phosphonic acid antibiotic		b	c					0	2	2	3	5	3
Pleuromutilin antibiotic			c					0	0	0	0	0	0
Rifamycin antibiotic	a		c			f		1	4	2	3	4	2
Streptogramin antibiotic			c					1	0	0	0	0	0
Sulfonamide antibiotic	a							0	1	0	0	0	0
Tetracycline antibiotic	a			d	e		g	1	4	5	5	5	5
**Total AR Gene Families**								**30**	**81**	**62**	**64**	**78**	**61**

Resistance mechanisms: a = antibiotic efflux, b = antibiotic inactivation, c = antibiotic target alteration, d = reduced permeability to antibiotic, e = resistance by absence (gene deletion), f = antibiotic target replacement, g = antibiotic target protection. *Antibiotic resistance

Bolded numbers represent column totals

CARD recognizes seven possible antibiotic resistance mechanisms (Table 3). Of these, efflux (38%) and target alteration (21%) were the most frequently occurring antibiotic resistance mechanisms across the set of oil fly isolates, though it was common for isolates to have multiple mechanisms of resistance for an individual drug class (Table 3). Target reduction and target protection accounted for less than 1% of the mechanisms associated with the oil fly isolates, and in both instances their presence occurred in combination with other mechanisms for the drug class; rifampicin resistance was associated with both efflux and target reduction, while tetracycline resistance was associated with both reduced permeability and absence of target. In the 12 instances where only a single mechanism was identified for a particular drug class, six were associated with efflux, and six were associated with target alteration.

Comparing the oil fly drug class results to the CARD Phenotype Prevalence Database reveals sixteen instances of apparently novel drug class hits, where the resistance elements targeting a particular drug class are present in the oil fly isolate but absent in the CARD database for the corresponding taxa (Table 4).

**Table 4:** CARD Prevalence Phenotype, listed for each of the 29 CARD drug classes observed in the oil fly isolate genomes. The CARD prevalence reflects the percent of genomes of the listed species that have been observed with resistance determinants in the listed class. Oil fly (OF) presence absence is listed for each isolate. A “ + + ” indicates that a determinant coding for resistance to that drug class was observed in the corresponding oil fly isolate, with “ + ” indicating instances where only 0–10% of the CARD isolates were observed to have determinants in the listed class. A “ε” indicates determinants from the listed drug class were not observed in the oil fly isolate. “nd” indicates that there was no data in the CARD database for this species

Antibiotic drug	*Alcaligenes Faecalis*		*Pseudomonas aeruginosa*		*Morganella Morganii*		*Providencia rettgeri*		*Providencia vermicola*		
	**CARD(%)**	**OF2**	**CARD(%)**	**OF4**	**CARD(%)**	**OF8**	**CARD(%)**	**OF6**	**CARD**	**OF5**	**OF10**
**Aminocoummarin**	0	** ++**	68	++	0	+	0	+	nd	++	++
**Aminoglycoside**	25	++	69	++	59	+	57	++	nd	++	++
**Bicyclomycin-like**	0	ε	67	++	0	ε	0	ε	nd	ε	ε
**Carbapenem**	17	++	69	++	59	++	56	++	nd	++	++
**Cephalosporin**	17	++	69	++	60	++	57	++	nd	++	+
**Cephamycin**	14	++	68	ε	57	++	57	++	nd	++	++
**Diaminopyrimidine**	10	ε	68	++	57	++	54	++	nd	++	++
**Disinfectants**	83	++	69	++	57	++	55	++	nd	++	++
**Elmafycin**	0	+	0	+	48	++	39	++	nd	++	++
**Fluoroquinolone**	78	++	69	++	59	++	54	++	nd	++	++
**Fusadane**	0	+	0	ε	0	ε	0	ε	nd	ε	ε
**Glycopeptide**	0	+	68	++	2	+	52	++	nd	++	++
**Glycylcycline**	0	ε	68	++	1	+	0	++	nd	ε	ε
**Isoniazid-like**	0	ε	0	ε	0	+	0	ε	nd	ε	ε
**Lincosamide**	2	ε	0	+	4	++	11	++	nd	++	++
**Macrolide**	5	+	68	++	58	++	54	++	nd	++	++
**Monobactam**	2	+	69	++	13	++	13	++	nd	++	++
**Nitroimidazole**	0	ε	0	ε	0	+	0	ε	nd	ε	++
**Nucleoside**	5	ε	0	ε	11	ε	21	ε	nd	ε	++
**Oxazolidinone**	0	+	68	++	0	+	1	+	nd	++	++
**Penam**	19	++	69	++	60	++	56	++	nd	++	++
**Penem**	14	++	69	++	56	++	55	++	nd	++	++
**Peptide**	0	+	68	++	57	++	52	++	nd	++	++
**Phenicol**	7	+	69	++	56	++	52	++	nd	++	++
**Phosphonic acid**	3	ε	67	++	56	++	52	++	nd	++	++
**Rifamycin**	0	+	68	++	9	+	53	++	nd	++	++
**Streptogramin**	2	+	1	ε	4	ε	14	ε	nd	++	++
**Sulfonamide**	29	ε	69	++	24	ε	29	ε	nd	ε	ε
**Tetracycline**	78	++	68	++	39	+	53	++	nd	++	++

CARD gene assignments (Supplementary Table S7) identified nine determinants that were common across all six isolates: *adeF*(multidrug efflux membrane fusion), *Escherichia coli* 23S rRNA with a mutation conferring resistance to erythromycin and telithromycin, *E. coli* EF-Tu mutants conferring resistance to Pulvomycin, *E. coli rpoB* mutants conferring resistance to rifampicin, *kdpE* (transcriptional activator), *Moraxella catarrhalis* 23S rRNA with a mutation conferring resistance to macrolide antibiotics, *Pasteurella multocida* 16S rRNA mutation conferring resistance to spectinomycin, and lastly a *Salmonella enterica* 16S rRNA (rrsD) mutation conferring resistance to spectinomycin. When comparing the oil fly assignments to the list of species-specific genes present in the CARD genotype database (see materials and methods for qualifications), eight of the nine common determinants had not previously been listed in the database for one or more of the oil fly isolate species. OF8 (*Morganella morganii*) and OF2 (*Alcaligenes sp.*) had the highest proportion of mapped reads that were not present in the CARD database, with 32% and 24% of mapped reads not matching the gene list for *Morganella morganii* and *Alcaligenes (faecalis)*, respectively. OF4 (*Pseudomonas aeruginosa*) had the largest diversity of genes, with 70 percent of the mapped reads from this study assigned to genes found only in that isolate, while OF6 (*Providencia rettgeri*) had only seven percent of mapped reads that were unique to that isolate.

## Discussion

One of the unique features of this study, compared to others that explore microbial life in La Brea and other asphaltene and polycyclic aromatic hydrocarbon-rich tar seeps (Schulze-Makuch, 2011; Kim and Crowley, 2007), is the examination of individual bacterial isolates. Pure-culture bacterial isolates allow for the conclusive linking of genotype and phenotype (Gill, 2017), and afford the opportunity to explore antibiotic resistance genes in the context of individual bacterial taxonomies.

Taxonomically, all six of the isolates characterized in this study were classified as Pseudomonata (nee proteobacteria). The lack of phylum-level diversity is a function of the high prevalence of Pseudomonata associated with hydrocarbons, including hydrocarbon-contaminated soil (Das et al. 2021; Cabral et al. 2022), and the culture techniques used in the original isolation (Kadavy et al. 1999). Oil fly larval gut isolates are subjected to two primary sets of environmental drivers—those provided by the heavy oil and asphalts of the tar pits, and those provided by the primary residence within the gut of the oil fly. The dipteran gut has been shown to be dominated by Pseudomonata (Sontowski and van Dam, 2020). Thus, the current set of isolates provides information on commonly found bacterial species relevant in both habitats. What is unique is not their taxonomy, but rather the profile of antibiotic resistance families represented in their genomes. Every isolate contained genes coding for at least one type of drug resistance not previously identified by the CARD phenotypic profile database for that species or genus (range 1–8).

### **Extremophiles as a source of antibiotic resistance genes**

The oil fly gut isolates examined in this study contain a core set of antibiotic resistance genes, as well as a suite of resistance families uncommonly found in these taxa. Extremophilic environments can spur the evolution and adaptation of bacterial communities (Li et al. 2014), allowing individual host bacteria to combat abiotic stressors (Bang et al. 2018). This accelerated evolution in response to stress has been highlighted as a way for environmental antibiotic resistance genes to develop (Kadavy et al. 2000; Bengtsson-Palme et al. 2018), particularly in the presence of hydrocarbons (Kadavy et al. 2000; Das et al. 2021). Our data provide further evidence in support of this premise.

The most commonly identified antibiotic resistance gene mechanism from the oil fly isolates in this study was efflux pumps (isolates coded for resistance to 22 drug classes with efflux mechanism). The primary efflux pumps, such as the ATP-binding cassette (ABC) found across all three kingdoms of life, function by creating an electrochemical gradient across the cell membrane. These efflux pumps then power secondary active member transporters such as the major facilitator super family (MFS), small multidrug resistance (SMR), and multidrug and toxic compound extrusion (MATE) transporters found in our oil fly isolates, imparting the ability to modulate an accessory set of efflux pumps that could be environment or substrate specific (Du et al. 2018; Lekshmi 2018). In other bacteria (Dinh et al. 1994; Nagakubo et al. 2002; Liu et al. 2022), efflux pumps extrude a variety of detergents (SDS, benzalkonium chloride, bile salts) and other noxious chemicals as well as antibiotics. The composition of the La Brea tar is highly weathered, meaning that few linear alkanes remain. TLC analysis of the tar showed 10% branched alkanes and cyclic alkanes, 47% aromatics, 30% resins, and 13% polars while GC/MS detected hopanes, phenanthrene, and C1, C2, and C3 phenanthrenes (Kadavy et al. 1999; Nickerson and Plantz, 2017). What would be more natural than finding polyaromatic hydrocarbon-extruding bacteria in the guts of oil fly larvae which are continuously ingesting tar/asphalt estimated to be 47% aromatic? Many of these compounds are highly toxic and/or mutagenic to bacteria. Oil fly bacteria, however, are resistant to both organic solvents and antibiotics. When pre-exposed to 20 µg of tetracycline per ml, seven of nine oil fly bacteria tolerated overlays of 100% cyclohexane, six of nine tolerated 10% xylene, benzene, or toluene (10:90 in cyclohexane), and three of nine (all *P. rettgeri*) tolerated overlays of 50% xylene—50% cyclohexane (Kadavy et al. 2000).

**Target site modification** is another common oil fly isolate resistance mechanism (resistance to 20 identified drug classes with this mechanism). Alteration of the target site for an antibiotic drug can limit the drug’s inhibitory or lethality functions. These alterations commonly occur via random point mutations in the core replication, transcription or translation machinery of a cell (Vrancianu et al. 2020). Elfamycin resistance determinants, identified in all isolates, was attributed exclusively to target site modification. Elfamycins work by blocking EF-TU, an elongation factor protein that catalyzes the binding of tRNA to the ribosome during translation (Prezioso et al. 2017). Based on data from the CARD phenotype prevalence database, this is the first instance of a *P. aeruginosa* isolate coding for elfamycin resistance. Our OF2 *Alcaligenes sp*. also coded for elfamycin resistance. In comparison, elfamycin resistance was not observed in the 20 Chromosomes, 5 plasmids, and 34 whole *A. faecalis* genomes screened for the CARD phenotype prevalence database. Elfamycin resistance is common in CARD database *Morganella* (48%) and *Providencia* (39%) genomes and was also identified in *Morganella* and *Providencia* isolated from the oil fly. In addition to what appears to be a novel appearance of elfamycin resistance in *P. aeruginosa*, target modification mechanisms are also attributed to the unusual presence of lacosamide resistance in *P. aeruginosa*, fusidane resistance in *Alcaligenes*, and isoniazid-like resistance in *M. morganii* from our oil fly isolates. Due to the oil fly life cycle, their gut bacteria are continuously exposed to hydrocarbons. Hydrocarbons cause oxidative stress to bacterial cells, leading to upregulation of the SOS response and mutagenesis (Sazykin and Sazykina, 2023), providing one explanation for the increase in target-site modification antibiotic resistance elements in these isolates via adaptive evolution.

### Antibiotic drug resistance in the oil fly gut

Fluoroquinolones and Aminoglycosides were the drug classes most frequently associated with resistance in the oil fly gut bacteria genomes, with a total of 44 and 36 resistance gene families identified across the six isolates, respectively. In addition to efflux and target modification, mechanisms for both types of drug resistance also include antibiotic inactivation. Based on CARD phenotypic prevalence data, fluoroquinolone resistance was common across the four genera represented by our isolates (range 55–78% of genomes containing fluroquinolone resistance genes). Studies in hydrocarbon-contaminated soils note a ten-fold increase in fluroquinolone resistance genes compared to the non-contaminated control soils (Das et al. 2021), consistent with the high number of fluoroquinolone resistance gene families identified in this study. Fluroquinolones work by targeting the DNA replication machinery and inducing the bacterial SOS stress response (Bush et al. 2020). Similarly, aminoglycosides act by disrupting protein synthesis (Lang et al. 2023). Adaption to life in the tar selects for bacteria with mechanisms that protect DNA and replication functions under mutational and oxidative stress. These same conditions may also contribute to selection of genetic elements observed in our isolates that similarly allow resistance to nucleic-acid-targeting antibiotics.

Of note is OF2, which could only be identified at the genus level. During our previous experiments (Kadavy et al. 1999; Kadavy et al. 2000; Nickerson et al. 2017), we identified a bacterial isolate that did not match existing databases at that time with regard to phenotypes (BBL Enterotube II, BBL, Cockeysville, MD; API 20E, bioMérieux, Durham, NC), or fatty acid composition (MIDI Labs, now Biolog, Hayward, CA). The results from the 16S rDNA sequence suggests that OF2 is an uncultured *Alcaligenes sp*. In the current experiment, the OF2 isolate contained genes to eight antibiotic drug classes not previously seen when compared with *Alcaligenes faecalis* isolates from the CARD database. Interpretation of this observation needs to take into consideration that the OF2 isolate could not be assigned to a species-specific taxonomy, and that the potential taxonomic difference of OF2 and *A. faecalis* may account for the apparent novelty of some drug classes identified in Table 4.

Some types of antibiotic resistance are postulated to have emerged from obscure genomes in a stressful environmental niche where they had been serving protective functions and ensuring homeostasis (Martinez et al. 2008; Aminov, 2009; Allen et al. 2010; Davies and Davies, 2010). A number of mechanisms that protect cells from antibiotic drugs can also protect cells from other environmental stressors, highlighting the functional physiological relevance of antimicrobial resistance genes in an environmental context (Aminov, 2009), providing one explanation of why these genes are enriched in our isolates in the absence of exposure to antibiotic drugs themselves, and adding to the evidence that AR arose from niche systems that attempt to combat life under stressful conditions. Likewise, there is robust evidence that exposure to environmental stresses (temperature, osmotic, pH) can lead to cross-protection to challenges by antibiotics (McMahon et al. 2007; Händel et al. 2016, Liao et al. 2020). For example, oil fly bacteria as a group require efflux pumps to survive in petroleum or environments contaminated with asphaltenes and other polyaromatic hydrocarbons, and these same efflux pumps also confer resistance to antibiotics. Although the presence of these resistance elements in the gut microbiome of the oil fly poses no direct threat to human health, there is evidence that clinically important resistance genes originate in environmental microbes (Kadavy et al. 1999; Kadavy et al. 2000; Wright, 2010). Concerns arise when genes that code for resistance are mobilized into new hosts, including agriculturally relevant plant and clinically relevant human and animal pathogens (Bengtsson-Palme et al. 2018; Peterson and Kaur, 2018), something that is facilitated by stressful conditions (Beaber et al. 2004; Schmidt et al. 2022; Wang et al. 2022), such as those in the tar pits. DNA sequencing data of the oil fly isolates in this study supports and strengthens earlier work suggesting survival under stressful environmental conditions contributes to the emergence of antibiotic resistance of human importance and provides evidence for the claim that “in nature there is more than one way to become resistant to antibiotics” (Kadavy et al. 1999).

## Conclusion

DNA sequencing revealed a novel combination of antibiotic drug resistance classes in the *H. petroli* larval gut isolates examined in this study. The primary mechanisms of antibiotic resistance coded for in the genome, including efflux pumps, target alteration, target inactivation, and reduced permeability, have ecologically and physiologically relevant functions that are likely selected for by life in the tar pits. These findings highlight the duality of functions performed by determinants that confer antibiotic resistance, and the need to conceptualize environmental antimicrobial resistance in the framework of bacterial ecology and the bacterial stress response. Continued exposure of oil fly gut bacteria to the tar preserves the pool of resistance genes identified here, in that niche. The antibiotic resistance profile of OF2 was unusual. While beyond the scope of this current evaluation, future work exploring the OF2 genome has the potential to elucidate the genetic potential of this novel isolate. All isolates are available for further research by other scientists via the NRRL https://nrrl.ncaur.usda.gov/. Finally, data presented here expands our fundamental understanding of the mechanisms, origin, and evolution of antibiotic resistance in extreme environments.

## Supplementary Information

Below is the link to the electronic supplementary material.

Supplementary file 1: OF2*Alcaligenessp* Mapped Reads

Supplementary file 2: OF4* Pseudomonas aeruginosa* Mapped Reads

Supplementary file 3: OF5* Providencia vermicola* 1 Mapped Reads

Supplementary file 4: OF6* Providencia rettgeri* Mapped Reads

Supplementary file 5: OF8* Morganella morganii* Mapped Reads

Supplementary file 6: OF10* Providencia vermicola* 2 Mapped Reads

Supplementary file 7: Gene Assignment from CARD Genotypic Database

## Data Availability

Data supporting the findings of this study are available within the paper and its Supplementary Information. The 16 s rRNA sequence accession numbers and WGS accession numbers are listed in Table 2. All 13 oil fly isolates were deposited into the Agricultural Research Service Northern Regional Research Laboratory (NRRL) United States Department of Agriculture bacterial culture collection in Peoria, IL. Strains are available for research purposes via https://nrrl.ncaur.usda.gov/.
